# Case report: Inflammatory bowel disease in Hermansky-Pudlak syndrome type 3 due to novel variant in HPS3

**DOI:** 10.3389/fgene.2025.1465527

**Published:** 2025-03-19

**Authors:** Jingqun Mai, Zhu Zhang, Bocheng Xu, Shanling Liu, He Wang, Hao Wang, Shuo Yang

**Affiliations:** ^1^ Department of Medical Genetics, West China Second University Hospital, Sichuan University, Chengdu, Sichuan, China; ^2^ Key Laboratory of Birth Defects and Related Diseases of Women and Children (Sichuan University), Ministry of Education, Chengdu, China

**Keywords:** Hermansky-Pudlak syndrome, HPS3, exome sequencing, inflammatory bowel disease, colitis, Glucocorticoids

## Abstract

**Background:**

Hermansky-Pudlak syndrome (HPS) is a rare autosomal recessive disorder with phenotypic and genetic heterogeneity, characterized by oculocutaneous albinism, bleeding diathesis, and other specific subtypes such as colitis. HPS3 is caused by biallelic mutations in *HPS3*. Patients with HPS3 have milder symptoms and were rarely reported to be involved in digestive disorders.

**Case summary:**

We report a case of an 11-year-old male patient who experienced chronic diarrhea and abdominal pain for a duration of 1 year, in the absence of identifiable predisposing factors. Colonoscopy and histopathological evaluations revealed extensive colonic inflammation characterized by erosion and lymphoid hyperplasia. Given the concurrent presence of albinism, horizontal nystagmus, and inflammatory bowel disease (IBD), molecular genetic testing was conducted, which is consistent with a diagnosis of Hermansky-Pudlak syndrome (HPS). Trio-based whole-exome sequencing (Trio-WES) identified a novel homozygous nonsense variant (NM_032383.5; c.2887G > T, p.E963*) in *HPS3*, leading to premature termination codons and aberrant splicing-mediated mRNA decay. The patient was treated with corticosteroids and mercaptopurine for management of IBD symptoms and has been attending follow-up appointments. Currently, the patient is in clinical remission; however, there remains a potential risk of relapse.

**Conclusion:**

We present a rare case of HPS-related IBD resulting from a homozygous variant in *HPS3* and provide insights into the understanding of the diagnosis and treatment of HPS3.

## Introduction

Hermansky-Pudlak syndrome (HPS) is a rare autosomal recessive disorder characterized by variable degrees of hypopigmentation, bleeding diathesis, nystagmus, and other diverse phenotypes such as colitis and pulmonary fibrosis. These manifestations result from multiple defects in lysosome-related organelles (LROs). In 1959, Frantisek Hermansky and Paulus Pudlak first described what is now known as Hermansky-Pudlak syndrome in two unrelated individuals presenting with bleeding diathesis associated with oculocutaneous albinism (Hermansky and Pudlak, 1959). To date, at least eleven causal genes associated with HPS have been identified in humans, classifying the syndrome into eleven distinct subtypes. These genes encode subunits of four obligate multi-subunit protein complexes: adaptor protein-3 (AP-3) and the Biogenesis of Lysosome-related Organelles Complex (BLOC)-1, BLOC-2, and BLOC-3 (Huizing et al., 2008b; Introne et al., 2000; Pennamen et al., 2020). AP-3 deficiency is associated with HPS2 (OMIM: 608233) and HPS10 (OMIM:617050). BLOC-1 deficiency includes HPS8 (OMIM:614077), HPS11 (OMIM:619172), HPS9 (OMIM:614171),and HPS7 (OMIM:614076). BLOC-2 deficiency encompasses HPS3 (OMIM:614072), HPS5 (OMIM:614074) and HPS6 (OMIM:614075). BLOC-3 deficiency is linked to HPS1 (OMIM: 203300), and HPS4 (OMIM: 614073). Overall, HPS-related genes include: *HPS1* (OMIM: 604982) (HPS1), *AP3B1* (OMIM: 603401) (HPS2), *HPS3* (OMIM: 606118) (HPS3), *HPS4* (OMIM: 606682) (HPS4), *HPS5* (OMIM: 607521) (HPS5), *HPS6* (OMIM: 607522) (HPS6), *DTNBP1* (OMIM: 607145) (HPS7), *BLOC1S3* (OMIM: 609762) (HPS8), *BLOC1S6* (OMIM: 604310) (HPS9), *AP3D1* (OMIM: 607246) (HPS10), and *BLOC1S5* (OMIM: 607289) (HPS11).

To our knowledge, HPS-related inflammatory bowel disease (IBD) primarily affects patients with BLOC-3 disorders (HPS-1, HPS-4) (Anikster et al., 2001; Introne et al., 2000). However, there is limited information available regarding IBD in patients with Hermansky-Pudlak syndrome type 3 (HPS3; OMIM:614072).

We herein present a rare case of HPS3 clinically manifested as chronic ulcerative colitis (UC). This report details the diagnostic process and treatment approach, with genetic testing playing a crucial role in confirming the final diagnosis.

## Methods

### Patient and informed consent

The study focused on an 11-year-old Chinese boy with HPS3. Written informed consent was obtained from all participants following standard ethical procedures, as approved by the Medical Ethics Committee of West China Second Hospital of Sichuan University.

### Clinical examination

Following the initial clinical evaluation, a comprehensive set of diagnostic tests was conducted, including colonoscopy, biopsy, laboratory tests (hematological analysis and fecal examination), abdominal computed tomography (CT), and chest X-ray radiography (XR).

### Whole-exome sequencing and data analysis

Peripheral blood samples (3–5 mL) were collected from the patient and his family in EDTA anticoagulated venous blood tubes, and genomic DNA was extracted from peripheral blood by using the QIAamp DNA Blood Mini Kit (Qiagen, Hilden, Germany). Trio-based whole-exome sequencing (Trio-WES) was performed by Nano WES Human Exome V2 (Berry genomics) in accordance with the manufacturer’s instructions. Exon-enriched libraries were sequenced through the NovaSeq6000 platform (Illumina) with 150 paired-end reads. Sequencing data was aligned to the human reference genome (GRCh38/hg38) via Burrows-Wheeler Aligner software (BWA). ANNOVAR and the Enliven Variants Annotation Interpretation System (Berry genomics) were employed for functional annotation. Candidate pathogenic variants related to the proband’s phenotypes were determined through comprehensive evaluation of related literature and disease databases, including ClinVar (http://www.ncbi.nlm.nih.gov/clinvar), PubMed (https://www.ncbi.nlm.nih.gov/pubmed/), OMIM (http://www.omim.org), HGMD (http://www.hgmd.org), etc.

Sanger sequencing was employed to validate candidate variants and conduct familial co-segregation analysis, thereby confirming Trio-WES results in all family members. Primer 6 software was utilized to design upstream and downstream primers, and specific PCR products from genomic DNA were analyzed on an ABI 3500xL Genetic Analyzer.

### RNA splicing analysis

To determine whether the variant resulted in aberrant splicing, RNA studies were conducted. Total RNA was isolated from peripheral blood mononuclear cells (PBMCs) using the Ultrapure RNA kit (CWBIO, Taizhou, China), and cDNA was synthesized with the RevertAid First Strand cDNA Synthesis Kit (Thermo, America). cDNA was amplified using primers designed with Primer Premier 6.0 to target *HPS3* from exon 15 to exon 17. Amplification products were subsequently resolved by electrophoresis in a 2.5% agarose gel, which was stained with ethidium bromide and photographed. The separated bands were then extracted from the agarose gel and analyzed using Sanger sequencing.

## Results

### Patient information and clinical features

**FIGURE 1:**
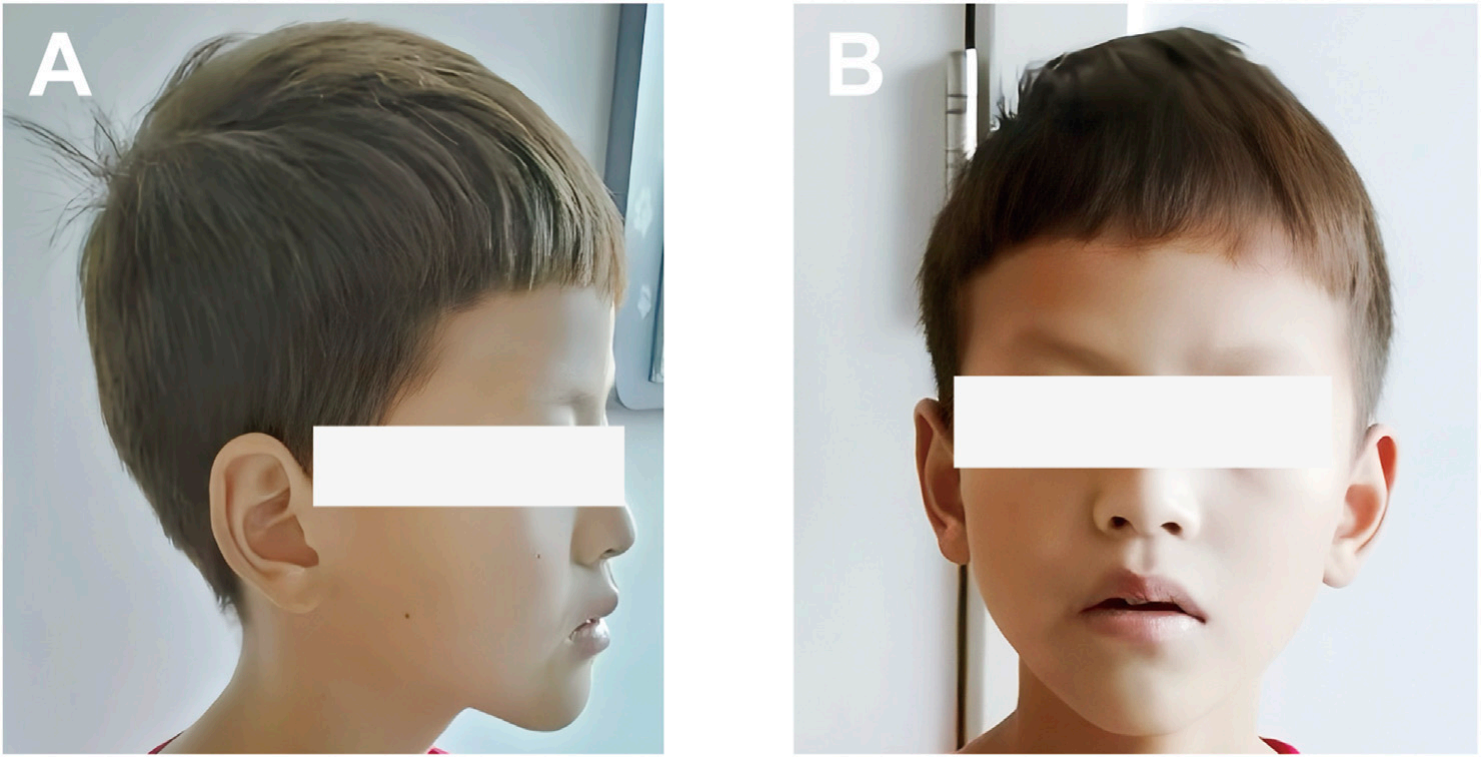
Photograph showing the skin and hair color of the patient: **(A)** Side view; **(B)** Front view. The patient presented mild hypopigmentation of the skin and hair.

An 11-year-old Chinese boy presented with a 1-year history of persistent diarrhea, occasionally bloody, accompanied by paroxysmal abdominal pain. He experienced approximately 5–6 bowel movements per day, characterized by loose stools, sometimes with blood. The abdominal pain was predominantly located in the left lower quadrant and upper abdomen, typically subsiding after defecation. In addition to colitis symptoms, he exhibited hypopigmentation of the skin and hair, horizontal nystagmus, and reduced visual acuity. On initial presentation, he was alert with stable vital signs. His height was 150 cm, and his weight was 30 kg. Physical examination revealed light brown hair and eyebrows, pale skin, horizontal nystagmus, and strabismus (Figures 1A,B). No rashes or petechiae were observed on the skin. The abdomen was soft, flat, and non-distended, without guarding or rebound tenderness. There were no significant abnormalities in intellectual, language, or motor development.

The patient had no significant past medical history apart from hypothyroidism, iron deficiency anemia, and undiagnosed congenital nystagmus and amblyopia. There was no relevant family history. He is the second-born child of healthy, unrelated Chinese parents with no known genetic diseases or maternal exposure to teratogens during pregnancy; both parents and the elder sister were in good health and did not exhibit similar conditions.

### Clinical findings

Laboratory testing revealed iron deficiency anemia, hypothyroidism, and thrombocytosis. The fecal occult blood test was positive. The stool-based Xpert MTB/RIF assay for *Mycobacterium tuberculosis* was negative. No pus cells or ova were detected in stool samples, and stool cultures (both aerobic and anaerobic) were negative, including for *Clostridium difficile*. Plasma CMV-DNA levels were below the detection threshold (<200 copies/mL). Based on these findings, infective enteritis was excluded from the differential diagnosis. Hemostatic parameters were within normal limits. The laboratory findings were shown in Table 1.

**TABLE 1:** Laboratory findings.

Testing item	Initial admission	Latest admission	Normal range
Hb (g/L)	78	123	110–146
MCV (fL)	62.4	78.3	77–92
MCHC (g/L)	280	310	310–355
ESR (mm/h)	55	55	<21
WBC (10^9^/L)	10.4	9.4	4.3–11.3
Neutrophils (%)	57.5	40.7	31–70
PLT (10^9^/L)	961	810	100–450
CRP (mg/L)	<0.5	16.2	0–8
TP (g/L)	71	67.5	62–76
ALB (g/dL)	40.8	42.1	38.0–54.0
T-BIL (μmol/L)	5.3	4.1	5–23
Cr (μmol/L)	58	51	35.9–83.1
Serum Iron (μmol/L)	1.60	5.90	10.6–36.7
Ferritin (ng/mL)	3.40	N/A	22–322
p-ANCA (IIF)	—	—	—
c-ANCA (IIF)	1:10 (+)	1:10 (+)	—
ANCA-PR3 (ELISA)	2.06	2.12	<1.0
ANCA-MPO (ELISA)	<1.00	<1.00	<1.0
TNF-α (pg/mL)	17.30	7.27	<8.1
IL2R (U/mL)	870.00	922.9	223–710

**FIGURE 2:**
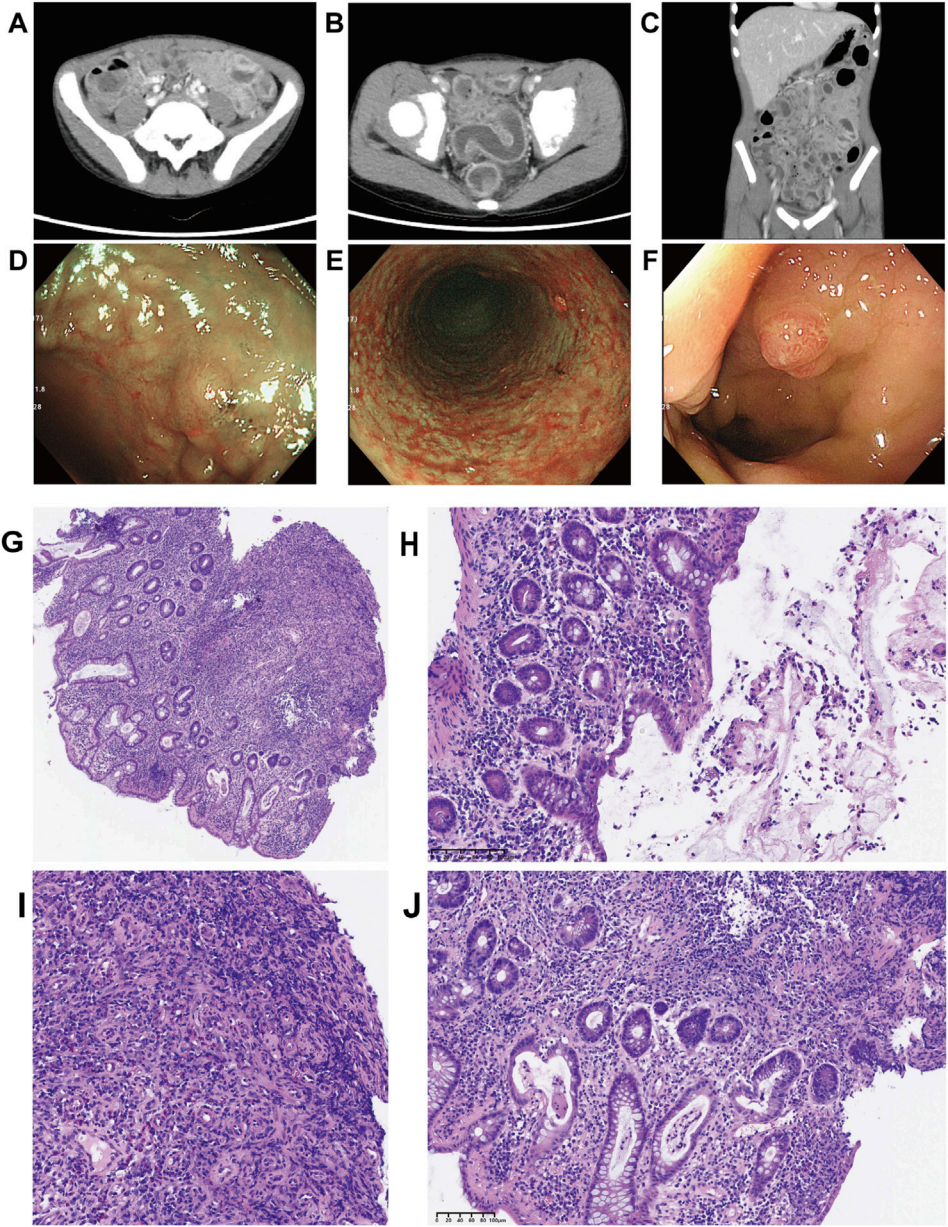
Computed tomography and colonoscopy findings upon the readmission to our hospital and Biopsy of sigmoid and rectal mucosa sections stained by HE. **(A–C)** The computed tomography images revealed bowel convergence of left upper quadrant, accompanied by wall thickening with contrast enhancement on the lumen side. Enlarged mesenteric lymph nodes were prominently visible; **(D–F)** Colonoscopy showed multiple superficial ulcers, erosions, nodular mucosa, and single rectal inflammatory polyp; **(G–J)** Pathological findings showed that crypt destruction, massive inflammatory infiltration of epithelium and lamina propria, crypt destruction, crypt distortion, crypt abscesses, crypt with branches, and granulation hyperplasia (**(G)** Low power view (400 μm), crypt distortion, crypt with branches, and granulation hyperplasia; **(H)** Medium power view (100 μm), mucous and suppurative exudate; **(I)** Medium power view (100 μm), granulation hyperplasia; **(J)** Medium power view (100 μm), Goblet cell depletion, crypt abscess).

CT revealed bowel convergence in the left upper quadrant, with wall thickening of the small intestine, colon, and rectum, accompanied by luminal contrast enhancement. Enlarged lymph nodes were observed in the colorectal mesentery, left obturator region, gastrocolic ligament, and gastropancreatic ligament (Figures 2A–C). Colonoscopy demonstrated multiple superficial ulcers, erosions, nodular mucosa, and edematous, congested, friable mucosa throughout the colon, predominantly in the transverse and left colon. An isolated inflammatory polyp was also identified in the rectum (Figures 2D–F). A chest X-ray showed no abnormalities.

Chronic non-atrophic gastritis was identified on biopsies obtained during upper gastrointestinal endoscopy. Hematoxylin and eosin (H&E) staining of mucosal biopsy specimens from the left colon, ascending colon, and ileocecal area revealed extensive acute and chronic inflammatory infiltration of the epithelium and lamina propria, crypt distortion, crypt abscesses, branched crypts, lymphoid hyperplasia, and granulation tissue hyperplasia (Figures 2G–J). These pathological findings provided strong supporting evidence for the diagnosis of IBD.

### Whole-exome sequencing and RNA splicing analysis

**FIGURE 3:**
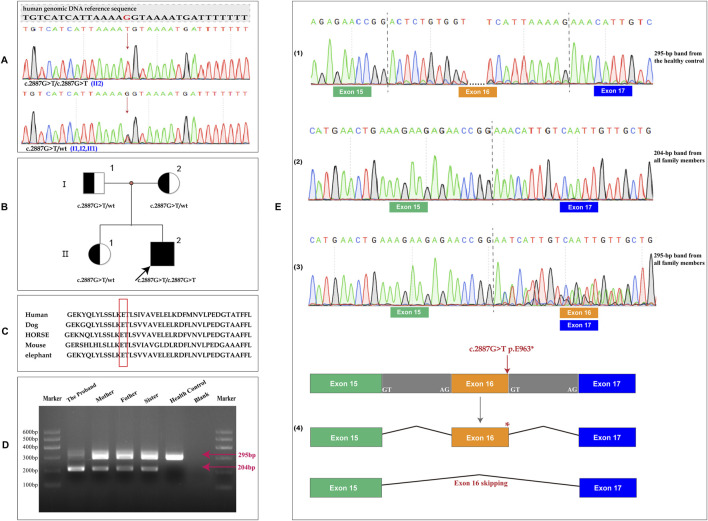
Genetic analysis of the family. **(A)** Sequencing of specific PCR products from genomic DNA revealed a homozygous variant in the *HPS3* gene (c.2887G > T, p.E963*) in the proband. The parents and the elder sister were found to be heterozygous carriers of this mutation.; **(B)** Pedigree of this family. Generations are denoted as I–II, with squares representing males and circles representing females. Filled symbols indicate affected individuals, and the arrow denotes the proband. **(C)** Multiple sequence alignment of the HPS3 homeodomain from different species of vertebrates showed the evolutionary conservation of the Glutamic Acid at position 963 (highlighted in red box); **(D)** Amplified exon 15 to 17 of *HPS3*, 2 *HPS3* cDNA products (295 bp and 204 bp) were identified in the proband and his family, but only 1 (295 bp) was identified in the healthy control; **(E)** Different RNA sequencing results in the proband, parents, sister and healthy control. (1) The 295-bp band in the healthy control exhibited a normal sequence. (2) Sequence analysis of the 204-bp band showed exon 16 skipping. (3) The 295-bp band in this family demonstrated two transcription products: one with exon 16 skipping and the other harboring the E963* mutation. (4) Pattern of biological effects of the c.2887G > T variant in the proband.

Based on the combination of albinism, horizontal nystagmus, and IBD, which were consistent with the clinical features of HPS, molecular genetic analysis was performed to confirm the diagnosis of HPS. Trio-WES analysis confirmed a homozygous nonsense variant (NM_032383.5; c.2887G > T, p.E963*) in the *HPS3* gene, inherited from heterozygous parents. The proband’s elder sister was identified as a heterozygous carrier of this mutation (Figures 3A,B). Sequence alignments of *HPS3* across species demonstrate the evolutionary conservation of the glutamic acid at position 963 (Figure 3C). This sequence change introduces a premature stop condon (p.E963*) in the *HPS3* gene and potentially disrupts the consensus sequence of the 5′ splice donor site of intron 16, which may result in an absent or disfunctional protein product. Bioinformatic analysis from franklin.genoox.com predicts that this variant may affect splicing (dbscSNV_ADA_SCORE: 1, dbscSNV_RF_SCORE: 0.99, and spliceAI_SCORE: 0.59) and suggests a classification of the variant as likely pathogenic. Therefore, RNA studies were conducted to evaluated whether the c.2887G > T variant caused aberrant splicing. Amplification of exons 15 to 17 revealed two *HPS3* cDNA products (295 bp and 204 bp) in this family, while only one product (295 bp) was observed in the healthy control. Sequence analysis indicated that the specific 204-bp band represented a sequence with exon 16 skipping (p.T933Kfs*10). In the proband and his family members, the 295-bp band corresponded to two transcriptional products: one with exon 16 skipping and the other harboring the E963* mutation. The healthy control showed only the normal transcript (Figures 3D,E). RNA studies identified a novel homozygous nonsense variant (c.2887G > T) in the *HPS3* gene, causing premature termination codons and mRNA decay, which might result in loss of gene function.

To date, this variant has not been reported in the literature on HPS3-related conditions and is absent from population databases such as 1,000 Genomes or gnomAD. According to the ACMG guidelines, this variant is classified as a variant of uncertain significance (VUS) (PVS1_Moderate + PM2_Supporting + PP4). In addition, Trio-WES analysis data revealed no other variations contributing to the index patient’s phenotypes in any potentially relevant genes, including *HPS1*.

### Final diagnosis

Molecular genetic analysis identified a homozygous nonsense variant in *HPS3* (c.2887G > T) that resulted in exon 16 skipping, leading to premature termination codons and aberrant splicing-mediated mRNA decay. These molecular changes confirmed the diagnosis of HPS3. Histological findings and medical history corroborated the presence of chronic UC, which is classified as an HPS-related IBD.

### Treatment

At the age of 11, the patient was admitted to our hospital for further evaluation and investigation. Based on the initial diagnosis of colitis, he was treated with mesalazine (150 mg/day), azithromycin (320 mg/day), marzulene, and various nutritional supplements, which alleviated his abdominal pain and diarrhea. Following discharge, he continued mesalazine therapy for 4 months. After 2 months, he was readmitted due to disease recurrence. He underwent a reevaluation via gastrointestinal endoscopes and received endoscopic resection of a solitary rectal inflammatory polyp. Meanwhile, trio-WES analysis was conducted on the patient and his parents, providing additional supporting evidence that the UC in this case was associated to HPS-related IBD. Due to the poor clinical response to the previous treatment protocol, adjustments were made. Cefoperazone-sulbactam in combination with metronidazole was administered to control infection, while enteral nutrition support was maintained using appropriate nutritional supplements. Methylprednisolone (20 mg BID) and other medications, including antithrombotic agents and levothyroxine sodium tablets, were subsequently initiated for comprehensive treatment. Symptoms of abdominal pain and diarrhea with bloody stool were significantly alleviated, leading to the patient’s discharge from the hospital. Within 4 months post-discharge, the patient underwent three endoscopic examinations, including endoscopic resection of rectal polyps. Subsequently, he was readmitted for further treatment due to slightly exacerbated diarrhea accompanied by paroxysmal fever. Upon admission, methylprednisolone was continued for anti-inflammatory purposes, and mercaptopurine (17 mg QD) was initiated concurrently for immune suppression. Following his discharge, the patient adhered to the prescribed medication regimen for 2 weeks but subsequently discontinued it independently. He was readmitted after 3 months due to a recurrence of diarrhea. The diagnosis and treatment protocol remained consistent with the previous regimen. The dosage of methylprednisolone was adjusted to 20 mg in the morning and 16 mg in the evening, with the addition of mesalazine. Following this adjustment, the patient’s diarrhea showed significant improvement. Since his discharge, he has been consistently taking methylprednisolone (18 mg BID). Currently, the patient is on mercaptopurine (25 mg BID) and methylprednisolone (20 mg QD), with planned weekly dose reductions until reaching a maintenance dose of 4 mg daily, along with ongoing adjustments during regular follow-up visits.

### Outcome and follow-up

The disease tends to recur frequently, resulting in four hospital admissions within a year. Corticosteroids have demonstrated greater efficacy compared to NSAIDs, providing superior symptom relief and supporting healthy growth. With treatment, the patient has gained 16 pounds in weight over 3 months, and colonoscopy findings show significant improvement in colitis. As of now, the prognosis appears favorable.

## Discussion

We report a rare case of HPS3 with the chief complaint of IBD caused by a novel homozygous variant in *HPS3* (OMIM: 606118; NM_032383.5; c.2887G > T, p.E963*).

HPS is characterized by oculocutaneous albinism, bleeding diathesis, and, in some individuals, other organ involvement in specific subtypes such as pulmonary fibrosis, granulomatous colitis, and/or immunodeficiency (Gahl et al., 1998; Huizing et al., 2008a). The human HPS subtypes (HPS1-HPS11) have different clinical features to some extent, whereas the disease spectrum is nearly identical in patients with mutations in different components of the same protein complex (Bowman et al., 2019; Dell’Angelica, 2004; Huizing et al., 2008b). Although the molecular function of BLOC-2 in LRO biogenesis is incompletely known, disorders such as pigmentation and bleeding are mildly affected in BLOC-2 (*HPS3*, *HPS5*, and *HPS6*) mouse models (Suzuki et al., 2001; Zhang et al., 2003), and most of the cases reported so far have comparably shown that HPS patients with BLOC-2 mutations tend to have a mild form of the disease, with no lung or immune involvement. Therefore, individuals with BLOC-2 deficiency can go undetected for decades (Ringeisen et al., 2013). To date, a total of 64 mutations of *HPS3* have been reported in the HGMD database. Molecular diagnosis of biallelic pathogenic (or likely pathogenic) variants in HPS-related genes with suggestive findings can clarify the genetic cause when clinical features are inconclusive (Asztalos et al., 2017; Ishihara et al., 2019; Wei et al., 2016). Similarly, the patient in our case presented typical HPS symptoms, such as mild degrees of light brown hair and eyebrows, pale skin, congenital horizontal nystagmus, and amblyopia. Due to the mild clinical features, he therefore remained undiagnosed until active colitis developed.

O’Brien et al. (2021) conducted a retrospective review of 261 HPS patients and found 37 cases of IBD (O’Brien et al., 2021). However, only five individuals with HPS3-related IBD were documented. These cases represented the sole documented occurrences of HPS3-related IBD. Though the precise phenotype of the patients and available treatment options have not been extensively documented, all patients had mild symptoms that required no treatment or less treatment. In our case, the main complaints were chronic diarrhea and abdominal pain. Clinical manifestations, colonoscopy, and histological findings confirmed ulcerative colitis, considered a form of HPS-related IBD. Fistulae and extra-intestinal manifestations had not developed yet. These findings were somewhat in line with the features of BLOC-2 disorder to a certain extent. By contrast, the boy in our study experienced four relapses within a year, presenting a more severe phenotype than the previously reported cases. He received medical treatment based on corticosteroids and mercaptopurine. Although the drugs may provide temporary relief for gastrointestinal symptoms following administration, he still has a high tendency for recurrence of IBD with the long-term medication of corticosteroids.

To date, there is no cure for HPS or its associated manifestations, and the present treatment methods are symptomatic treatment to reduce complications (Asztalos et al., 2017). Treatment for HPS-related IBD resembles those for Crohn’s disease and ulcerative colitis, but responses to therapy are variable in patients with HPS, many of whom presented poor clinical response to corticosteroids and had to be treated with anti-TNF alpha biologic drugs (Felipez et al., 2010; Hazzan et al., 2006; Kouklakis et al., 2007). Infliximab, a chimeric monoclonal antibody directed against TNF-α, has been approved as a biologic agent for treatment of inflammatory and fistulizing Crohn’s disease. Several studies showed that anti-TNF-α drugs still have variable efficacy for HPS-related IBD (Erzin et al., 2006; Felipez et al., 2010; Grucela et al., 2006; Kouklakis et al., 2007; Yoshiyama et al., 2009). In the study of O’Brien, K. J. et al. (2021), 40% of patients with HPS-related IBD received biologic drugs due to their poor response to corticosteroids or non-steroidal immunosuppressants. More than half of patients who received anti-TNF alpha drugs experienced prolonged clinical response compared to a rate of approximately 30% of patients with non-syndromic IBD (Siegel and Melmed, 2009). While there is currently no available data on the use of anti-TNF-α drugs for treating HPS3-related IBD, these biologic drugs or even surgical interventions must be considered in our case to prevent severe complications if the clinical response to the current treatment goes worse and the disease progresses faster.

Genetic analysis of the patient and his family by Trio-WES revealed a novel homozygous mutation in *HPS3* that could explain the patient’s phenotype. This variant creates a premature translational stop signal (p.E963*) in *HPS3*, which is in the last base of the penultimate exon, leading to a deletion of 42 amino acids (4.1% of the total *HPS3* protein). Meanwhile, this variant could also result in aberrant splicing via disrupting the consensus sequence of the 5′ splice donor site of intron 16, which is expected to result in aberrant-splicing-mediated mRNA decay. Since the variant was located in the final base of the penultimate exon, it was not expected to trigger nonsense-mediated decay. The variant would result in a removal of less than 10% of the protein. Therefore, we decreased PVS1 by two levels (PVS1_M). This variant is not present in the general population databases such as 1,000 Genomes or gnomAD (PM2_Supporting). The patient presented typical symptoms of HPS3, which had little overlap with other clinical presentations, providing supportive evidence of pathogenicity (PP4). According to the ACMG guidelines, this variant is defined as that of uncertain significance, based on the above evidence (PVS1_M, PM2_P, and PP4). This LoF mutant is the one located at the very end of the coding regions among all the currently known LoF mutants. Despite assessing the target mutant as a variant of uncertain significance (VUS), combined with the clinical phenotypes of the patient, we still tend to regard it as the genetic cause that leads to HPS-related symptoms. Meanwhile, considered the more severe IBD phenotype in this case compared to the effects of the LoF variant, the pathogenesis of HPS3-related IBD and the distribution of IBD according to HPS genotypes need further study.

At present, cases of HPS3-associated IBD were infrequently reported, with only five patients documented in a retrospective single-centre cohort study that lacked detailed phenotypic descriptions and specific treatment regimens. Herein, we presented a case of adolescent-onset HPS3 with IBD as the initial symptom, detailing comprehensive clinical, imaging, pathological, and genetic features. Additionally, we identified a novel variant in *HPS3* that expanded the spectrum of HPS3-related IBD mutations. The patient’s IBD symptoms exhibited a transient response to corticosteroid therapy, but recurrence remains a concern. Long-term follow-up will be conducted to monitor his condition.

## Conclusion

Overall, we presented a rare case of HPS3-related IBD characterized by more severe ulcerative colitis symptoms than previously reported. This case included a comprehensive description of clinical features and treatment protocols. The identification of a novel variant in *HPS3* expanded the spectrum of HPS3-related IBD mutations.

## Data Availability

The variation data reported in this paper have been deposited in the Genome Variation Map (GVM) in National Genomies Data Center, Beijing Institute of Genomics. Chinese Academy of Sciences and China National Center for Bioinformation, under accession number GVM000983 (https://ngdc.cncb.ac.cn/gvm/).
